# Characteristics of corticomuscular coupling during wheelchair Tai Chi in patients with spinal cord injury

**DOI:** 10.1186/s12984-023-01203-x

**Published:** 2023-06-17

**Authors:** Yangmin Zu, Lina Luo, Xinpeng Chen, Haixia Xie, Chich-Haung Richard Yang, Yan Qi, Wenxin Niu

**Affiliations:** 1 Shanghai YangZhi Rehabilitation Hospital (Shanghai Sunshine Rehabilitation Center), School of Medicine, Tongji University, Shanghai, China; 2 Laboratory of Biomechanics and Rehabilitation Engineering, School of Medicine, Tongji University, Shanghai, China; 3 Department of Physical Therapy, College of Medicine, Tzu Chi University, Hualien City, Taiwan; 4 Sport Medicine Center, Hualien Tzu Chi Hospital, Buddhist Tzu Chi Medical Foundation, Hualien City, Taiwan; 5 Department of Rehabilitation Sciences, Tongji University School of Medicine, Shanghai, China

**Keywords:** Spinal cord injury, Tai Chi, Surface electromyography, Functional near-infrared spectroscopy, Corticomuscular coupling

## Abstract

**Background:**

Wheelchair Tai Chi (WCTC) has been proved to have benefits for the brain and motor system of spinal cord injury (SCI) patients. However, the characteristics of corticomuscular coupling during WCTC are scarcely known. We aimed to investigate changes following SCI on corticomuscular coupling, and further compare the coupling characteristics of WCTC with aerobic exercise in SCI patients.

**Methods:**

A total of 15 SCI patients and 25 healthy controls were recruited. The patients had to perform aerobic exercise and WCTC, while healthy controls needed to complete a set of WCTC. The participants accomplished the test following the tutorial video in a sitting position. The upper limb muscle activation was measured from upper trapezius, medial deltoid, biceps brachii and triceps brachii with surface electromyography. Cortical activity in the prefrontal cortex, premotor cortex, supplementary motor area and primary motor cortex was simultaneously collected by functional near-infrared spectroscopy. The functional connectivity, phase synchronization index and coherence values were then calculated and statistically analyzed.

**Results:**

Compared to healthy controls, changes in functional connectivity and higher muscle activation were observed in the SCI group. There was no significant difference in phase synchronization between groups. Among patients, significantly higher coherence values between the left biceps brachii as well as the right triceps brachii and contralateral regions of interest were found during WCTC than during aerobic exercise.

**Conclusion:**

The patients may compensate for the lack of corticomuscular coupling by enhancing muscle activation. This study demonstrated the potential and advantages of WCTC in eliciting corticomuscular coupling, which may optimize rehabilitation following SCI.

**Supplementary Information:**

The online version contains supplementary material available at 10.1186/s12984-023-01203-x.

## Background

Spinal cord injury (SCI) is a serious injury of central nervous system (CNS), and its prevalence is about 180,000 cases and increasing with an annual incidence of 23.7 cases per million population worldwide [1]. The injury has devastating physical implications and requires lifelong rehabilitation [2]. These patients are often confined to a sitting position and nearly half of them rely on wheelchairs to get around [3].

Exercise after SCI has been proved to improve functional prognosis and induce cerebral cortex recombination. Through performing physical activity, the patients can improve their exercise capacities and expand the sensorimotor cortex area associated with the muscle tissue above the damaged region [4]. Functional improvement after exercise is related to the degree of activation of the motor cortex [5].

Wheelchair Tai Chi (WCTC) which is also called seated Tai Chi, with its simple and easy to learn, slow movement characteristics, meets the need to improve exercise options for SCI patients [6]. Many studies have been carried out on WCTC. Our previous works have confirmed that it is a feasible, safe and effective exercise [7, 8].

WCTC has excellent potential and advantages in eliciting brain function and executive control ability. Previous studies have shown that WCTC can alter the functional network plasticity and gray matter volume to improve cognitive function [9, 10]. Using the oxygenation level obtained with near-infrared spectroscopy, Tsang et al. [11] explored the underlying brain functional mechanisms of WCTC in improving cognition and reducing the risk of falls, which could improve motor performance in dual tasks, and increase prefrontal activation and functional connectivity.

The motor tasks not only depend on good muscle function and sensory feedback, but also closely related to the CNS [12], which needs to design commands to regulate multiple muscles to complete various complex multi-joint cooperative movements with specific patterns and activation time [13]. In the process of autonomic movement, there is automatic synchronization between certain areas of the cerebral cortex and the peripheral nerves associated with muscle tissue. Corticomuscular coupling refers to the phenomenon that the cerebral cortex sends out control information, and sends movement instructions to the muscles through the spinal motor neurons, which causes synchronous oscillation of the cortex and the corresponding neuromuscular tissues [14]. Previous studies have made successful attempts to explore the coupling relationship. The coherence values of surface electromyography (sEMG) and electroencephalogram signals in different frequency bands can reflect the relevant information of the coupling between cortex and peripheral muscles [15]. To our knowledge, there is a lack of corticomuscular coupling studies on WCTC.

The sEMG signals are derived from the bioelectrical activity of spinal motor neurons under the control of the cortex [16]. Functional near-infrared spectroscopy (fNIRS) is a non-invasive brain imaging technique based on the principle of neurovascular coupling [17]. It has a relatively high temporal resolution and robustness for motion and has been widely adopted to investigate cortical responses during motor tasks [18]. As WCTC is more focused on the interaction between CNS and motor system than aerobic exercise (AE), the question of whether WCTC’s effect on the corticomuscular coupling is superior to AE will hopefully be answered.

Therefore, the aim of the present study was to investigate changes following SCI on corticomuscular coupling, and further compare the coupling characteristics of WCTC with AE in SCI patients. We hypothesized that (1) SCI may lead to diminished corticomuscular coupling due to the damaged neural circuits. (2) WCTC is more conducive to enhancing corticomuscular coupling than AE. The results of this study can be used to develop recommendations for the rehabilitation of SCI.

## Methods

### Participants

A total of 15 patients and 25 healthy subjects were recruited from Shanghai YangZhi Rehabilitation Hospital (Table 1). All participants were right-handed, able to communicate and follow instructions, and had experience of Tai Chi. Patients met the diagnostic criteria for SCI according to the American Spinal Injury Association [19] and were able to sit independently for more than 30 min. The exclusion criteria were: (1) history of musculoskeletal diagnosis; (2) neurological disorders or other chronic diseases; (3) visual or auditory impairments.

**Table 1:** Characteristics of the participants

	SCI group (n = 15)	HC group (n = 25)
Age, years	33.98 ± 6.10	25.33 ± 1.02
Gender (male/female), n	14/1	17/8
BMI, kg/m^2^	21.29 ± 3.85	21.93 ± 2.08
Duration of injury, years	10.00 ± 3.18	NA
Injury level		NA
T2–T5	1	
T6–T12	11	
Below L1	3	

Values are mean ± SD

This study conformed to the ethical requirements of the Helsinki Declaration. Ethical approval for the protocol was obtained from the ethics committee of the hospital. Written informed consent was obtained from each participant prior to the study.

### Experimental design and procedure

The demographic characteristics of participants were recorded in advance. SCI group was required to attend two sessions which were WCTC and AE. AE used in this study is a regular rehabilitation exercise for patients with SCI. The type of exercise in the first session was determined by lottery, and the other exercise was tested 1 week later. HC group was asked to complete a set of WCTC. Participants were required to watch a video of WCTC/AE, which lasted for 1 min and 50 s, and then to take a 10-min practice. All subjects were ensured to perform correct movements.

**Fig. 1:**
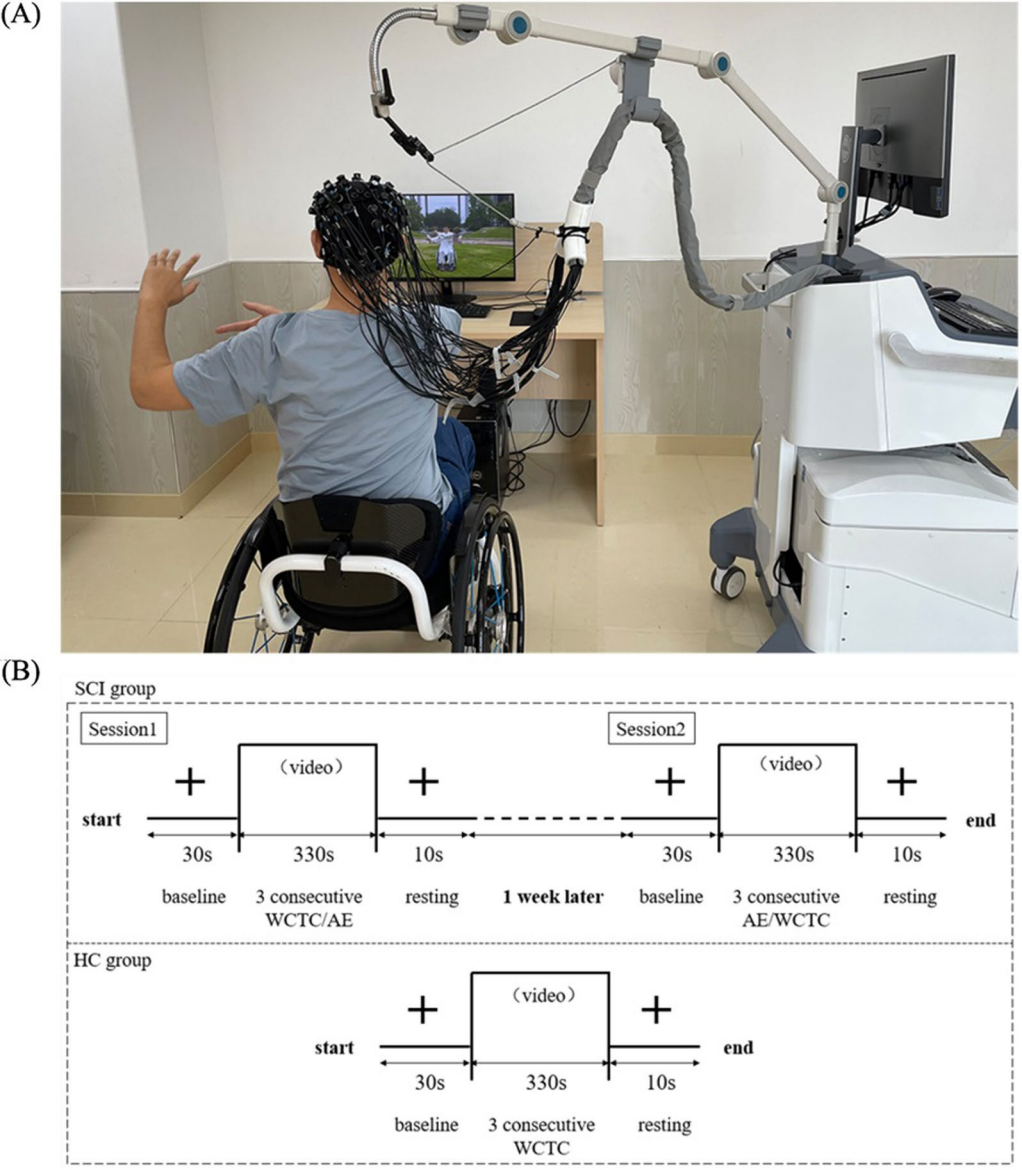
The experimental design. **A** Experimental setup and **B** experimental procedure

During data collection phase, subjects were instructed to take a 30-s rest, then to follow the video to complete 3 consecutive WCTC/AE (totally 330 s), and ended with a 10-s rest (Fig. 1). During rest periods, participants were instructed to focus on a cross displayed on a computer screen. A computer voice prompted the start and end of the test. The sEMG and fNIRS signals were recorded during the process. The current experiment was conducted in a quiet room to avoid environmental disturbances.

### fNIRS data acquisition and processing

**Fig. 2:**
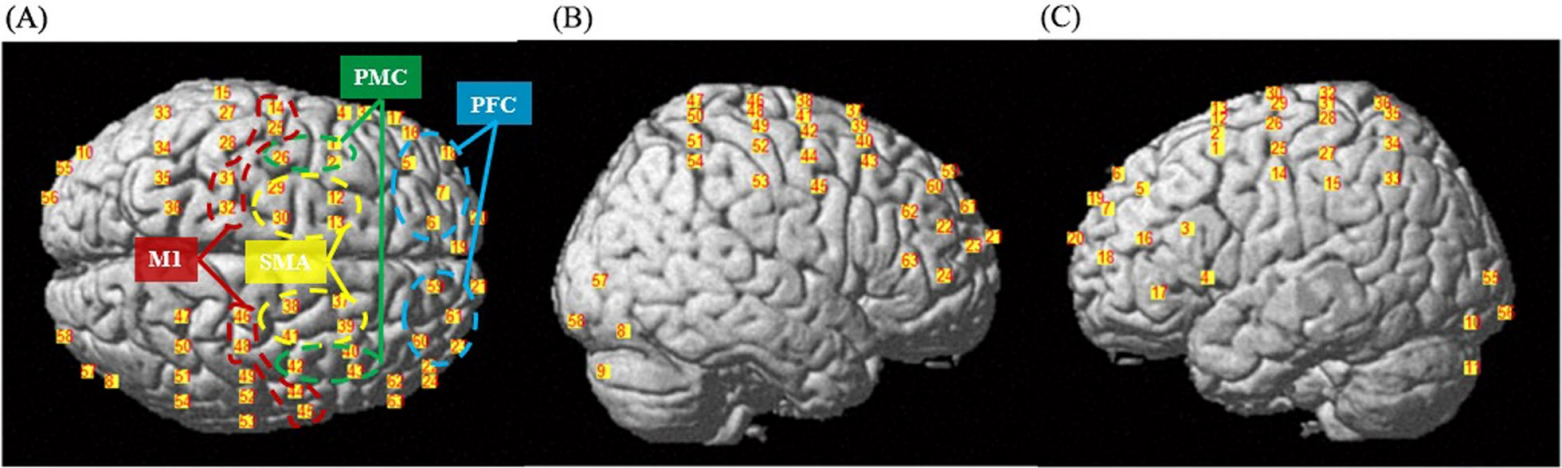
Arrangement of the fNIRS channels. **A** Top, **B** right-lateral and **C** left-lateral views of the locations of the channels in the MNI brain

Cortical activity was recorded by an fNIRS system (NirScan, Danyang Huichuang Medical Equipment Co., Ltd., China) with wavelengths of 740 nm and 850 nm at a sampling rate of 10 Hz. The optical system consisted of 24 emitters and 24 detectors, with a 3-cm inter-optode distance. The configuration of these sources and detectors provided 63 measurement channels. The probe placement was illustrated in accordance with the International 10–20 system (Fig. 2). The position of Cz was at the intersection of CH 30, 32, 38 and 46. A 3D position-measuring system (FASTRAC; Polhemus, Colchester, VT, USA) was used to locate the probes. The obtained coordinates were then transformed into the Montreal Neurological Institute (MNI) coordinates using the NIRS-SPM toolbox [20]. The cortical projection coordinates of all channels in MNI space were identified (Additional file 1: Table S1).

Based on the estimated spatial registration on MNI, eight regions of interest (ROIs) were selected. A probabilistic registration approach was used for mapping the channels for each ROI [21]. In this study, we quantified the activity changes in the prefrontal cortex (PFC, left: CH 5, 6, 7 and 18, right: CH 23, 59, 60 and 61), premotor cortex (PMC, left: CH 1, 2 and 26, right: CH 40, 42 and 43), supplementary motor area (SMA, left: CH 12,13, 29 and 30, right: CH 37, 38, 39 and 41) and primary motor cortex (M1, left: CH 14, 25, 31 and 32, right: CH 44, 45, 46 and 48).

The fNIRS data were preprocessed using the open-source package HomER2 [22]. The raw signals were converted to optical density changes, then a spline interpolation algorithm was performed to correct motion artifacts caused by head movements [23]. Then, the corrected signals were bandpass filtered to 0.01–0.1 Hz to remove baseline drift and physiological noises including heart rate. The filtered optical density data were finally used to derive the relative concentration changes in oxyhemoglobin (HbO_2_) and deoxyhemoglobin (HbR) based on the modified Beer-Lambert Law [24]. Hemodynamic response function (HRF) was obtained after block averaging, by subtracting the mean of the baseline (the last 5 s of the rest phase) from the mean value during the plateau of the response (from 10 to 100 s after onset). Considering concentration changes in HbO_2_ (ΔHbO_2_) are more sensitive to regional cerebral blood flow due to higher signal-to-noise ratios, we adopted ΔHbO_2_ as a HRF indicator for further analyses [25].

Functional connectivity (FC) between ROIs was evaluated systematically based on spontaneous oscillation of ΔHbO_2_. The FC between channels was defined as the Pearson correlation coefficient between the changes in HbO_2_ levels in each channel [26]. The Fisher Z transformation was applied to acquire a z-value for individual correlation coefficient. Then the average value of the correlation coefficients was yield using inverse Fisher transformation. The mean FC between ROIs was obtained by averaging the correlation coefficients of all channel pairs in these two regions.

### sEMG data acquisition and processing

Muscle activity was recorded at 2000 Hz using a wireless EMG telemetry system (Noraxon Inc. Scottsdale, USA) from the following muscles on both sides: upper trapezius (UT), medial deltoid (MD), biceps brachii (BB) and triceps brachii (TB). Electrodes were placed at target positions according to SENIAM recommendations [27]. The collected signals were synchronized with the fNIRS system.

All sEMG data were processed in MATLAB (MathWorks, USA). Raw data were bandpass filtered at 10–400 Hz using a second-order Butterworth filter, then full-wave rectification was performed. The obtained signals were set with a window length of 100 ms to calculate the root mean square (RMS). The normalized RMS amplitude was finally acquired by the maximal voluntary contraction (MVC) method.

### Corticomuscular coupling

On the basis of brain and muscle activation analysis, the average ΔHbO_2_ within each ROI and normalized RMS were acquired. In time domain and frequency domain, phase synchronization index (PSI) and coherence value were calculated respectively to quantify corticomuscular coupling.

#### Phase synchronization

Phase synchronization analysis is used to analyze two or more signals oscillating periodically in a repetitive sequence of phase angles [28]. It examines the relationship of instantaneous phase between signals but neglecting the influence of amplitudes [29]. The calculation formula of phase synchronization index (PSI) is defined as:

1 $$\begin{array}{c}\text{PSI}=\sqrt{{\langle \mathrm{cos}{\theta }_{xy}^{H}\left(t\right)\rangle }_{t}^{2}+{\langle \mathrm{sin}{\theta }_{xy}^{H}\left(t\right)\rangle }_{t}^{2}}\end{array}$$

2 $$\begin{array}{c}{\theta }_{xy}^{H}\left(t\right)=n{\theta }_{x}^{H}\left(t\right)-m{\theta }_{y}^{H}\left(t\right)\end{array}$$

where ${\theta }_{x}^{H}\left(t\right)$ and ${\theta }_{y}^{H}\left(t\right)$ are phase angles of filtered sEMG and fNIRS signals after the Hilbert transform, m and n are taken as 1. PSI is between 0 and 1.

#### Coherence analysis

Coherence analysis is a method to describe the degree of similarity between two signals in the frequency domain. With reference to Terry and Griffin’s research, the segment length was set to 100 sample points, the overlap was set to 50%, and the short-time Fourier transform segment taper was Hanning window [30]. The estimation of coherence can be expressed by:

3 $$\begin{array}{c}{C}_{xy}=\frac{{\left|{P}_{xy}\left(f\right)\right|}^{2}}{{P}_{xx}\left(f\right)*{P}_{yy}\left(f\right)}\end{array}$$

where $f$ is the frequency, ${P}_{xy}\left(f\right)$ is the cross-spectrum between sEMG and fNIRS signals, ${P}_{xx}\left(f\right)$ and ${P}_{yy}\left(f\right)$ are the averaged auto spectra of sEMG and fNIRS signals, respectively. The coherence value is in the range of 0–1. A value close to 0 indicates weak coherence, whereas a value close to 1 indicates strong coherence [31].

The sEMG and fNIRS data from 30 to 90 s after onset were selected to compute the PSI and coherence values between one ROI and the contralateral muscle for each subject. Then we averaged the above values across subjects as the group-level values.

### Statistical analysis

Statistical analyses were performed using SPSS 25 (IBM SPSS Statistics, Chicago, IL, USA). The normality of data was verified by means of Shapiro–Wilk test. Chi-square and independent *t*-tests were used to examine the homogeneity of the demographic data between groups. Independent *t*-tests and paired *t*-tests were carried out separately to evaluate intergroup differences during WCTC as well as to analyze any difference caused by the form of exercise among patients with SCI, regarding cortical activity, muscle activity and corticomuscular coupling. The statistical significance was set at two-tailed *P* < 0.05. Furthermore, *t*-statistic maps computed for group analysis were plotted onto a conventional brain template by the BrainNet Viewer toolbox.

## Results

### Cortical activation and functional connectivity

**Fig. 3:**
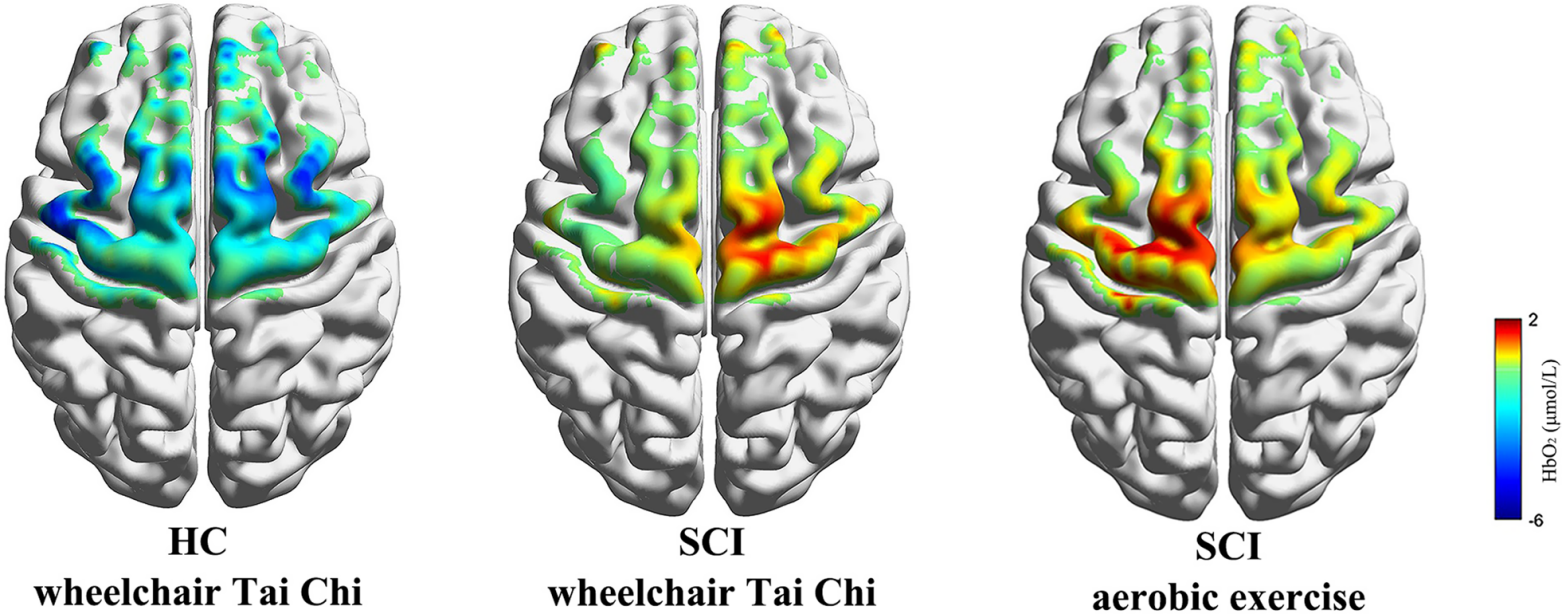
Concentration changes of oxyhemoglobin (ΔHbO_2_) in HC (during WCTC) and SCI (during WCTC and AE) group

**Fig. 4:**
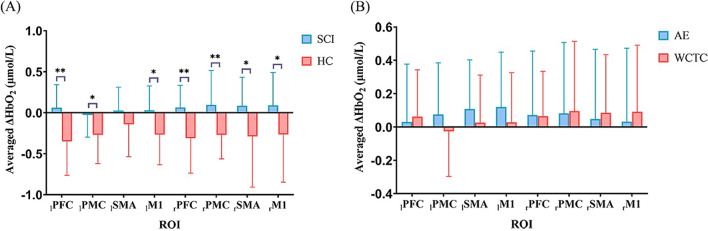
The significant differences of cortical activation (**A**) between SCI and HC group during WCTC (**B**) between AE and WCTC among SCI group in both hemispheres. *P < 0.05, **P < 0.01; PFC: prefrontal cortex; PMC: premotor cortex, SMA: supplementary motor area; M1: primary motor cortex. l: left upper limb; r: right upper limb

The mean ΔHbO_2_ values during WCTC in HCs as well as during WCTC and AE in patients with SCI are shown in Fig. 3. The results with significant differences are shown in Fig. 4. The _l_PFC (*P* = 0.002), _r_PFC (*P* = 0.004), _l_PMC (*P* = 0.027), _r_PMC (*P* = 0.002), _l_M1 (*P* = 0.012), _r_M1 (*P* = 0.043) and _r_SMA (*P* = 0.038) of SCI patients had greater activation than HCs. No significant difference was found between WCTC and AE in terms of the ΔHbO_2_.

**Fig. 5:**
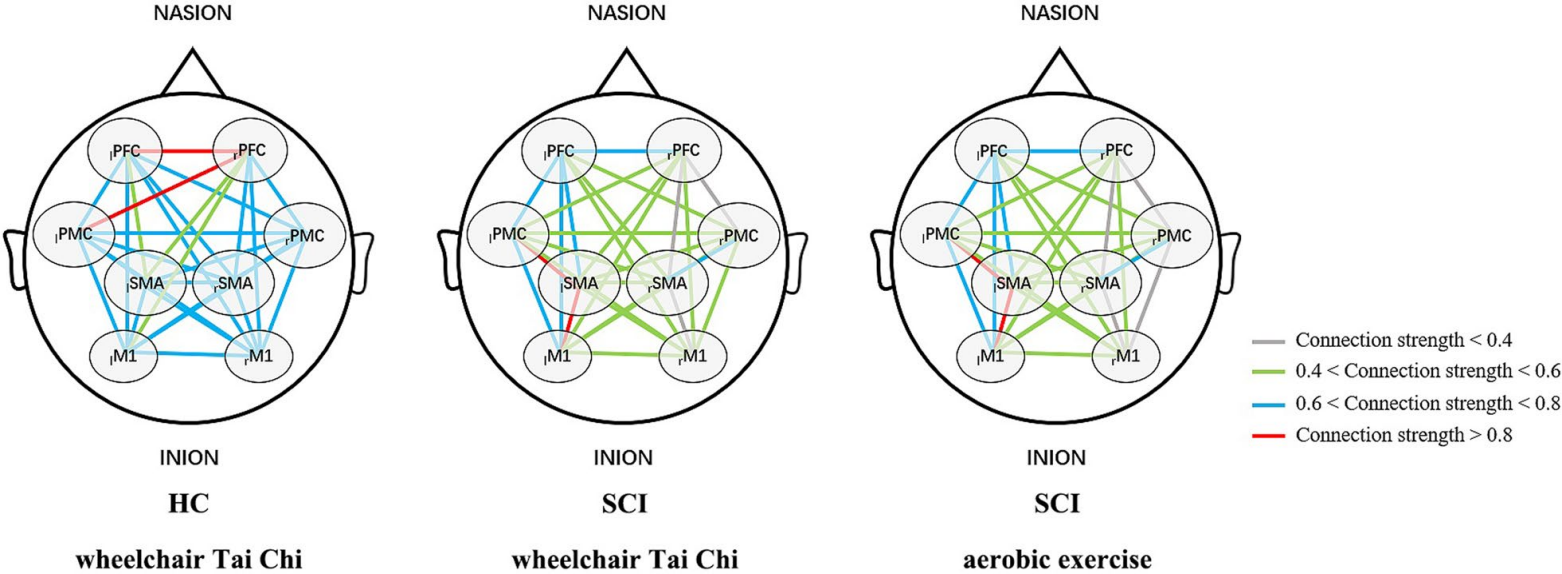
Correlation analysis based on ΔHbO_2_ of cortex in HC (during WCTC) and SCI (during WCTC and AE) group. Line color indicates the connectivity intensity between two ROIs

**Fig. 6:**
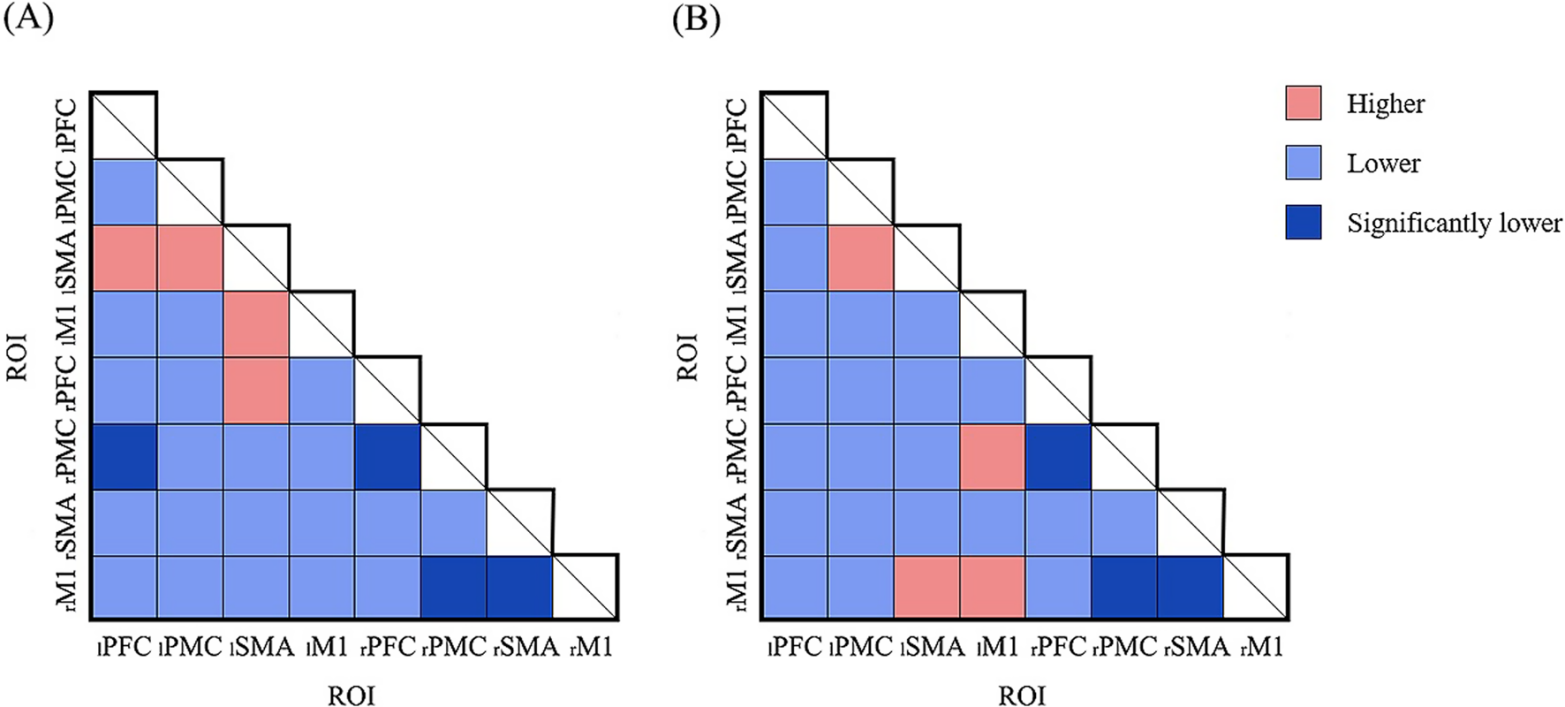
Comparison of FC values (**A**) between SCI and HC group during WCTC (**B**) between AE and WCTC among SCI group. Red indicates the increased FC, while blue indicates the decreased FC. Dark blue indicates the significance (*P* < 0.05)

Figure 5 shows the connectivity maps during WCTC in HCs as well as during WCTC and AE in patients, and provides a visual indication of the connectivity among the cerebral regions. Figure 6A shows the significant differences of FC values between patients, and HCs and Fig. 6B shows the significant differences between WCTC and AE in patients. The connectivity of _l_PFC-_r_PMC (*P* = 0.044), _r_PFC-_r_PMC (*P* = 0.004), _r_PMC-_r_M1 (*P* = 0.029) and _r_SMA-_r_M1 (*P* = 0.001) in HCs was stronger than that in patients. Moreover, in the SCI group, AE showed significantly higher _r_PFC-_r_PMC (*P* = 0.038), _r_PMC-_r_M1 (*P* = 0.003) and _r_SMA-_r_M1 (*P* = 0.014) compared with WCTC. On the whole, FC in SCI patients was decreased compared with HCs and WCTC also showed an overall decrease in FC compared with AE among patients.

### Muscle activation

The results with significant differences are shown in Table 2. The activation of both sides of BB (left: *P* = 0.003, right: *P* = 0.009) in the SCI group were greater than that in the HC group. Both sides of BB (*P* < 0.001) and TB (*P* < 0.001) had greater activation during AE than during WCTC.

**Table 2:** The significant differences of muscle activation

	WCTC			SCI group		
	SCI group	HC group	*P* value	AE	WCTC	*P* value
_l_UT	28.93 ± 18.28	22.42 ± 19.55	0.371	50.90 ± 20.09	41.37 ± 21.11	0.197
_l_MD	13.64 ± 12.51	7.59 ± 3.54	0.164	12.60 ± 12.02	11.90 ± 10.85	0.630
_l_BB	16.06 ± 8.31	5.53 ± 3.26	0.003*	23.41 ± 15.13	15.21 ± 11.02	< 0.001*
_l_TB	5.30 ± 3.20	3.79 ± 2.73	0.168	16.07 ± 7.75	5.40 ± 2.95	< 0.001*
_r_UT	27.60 ± 20.08	26.33 ± 18.27	0.857	25.96 ± 11.66	29.72 ± 16.71	0.061
_r_MD	15.49 ± 9.43	12.99 ± 6.57	0.378	11.24 ± 5.08	13.79 ± 8.20	0.065
_r_BB	11.58 ± 6.35	4.83 ± 3.13	0.009*	21.66 ± 9.07	11.66 ± 7.00	< 0.001*
_r_TB	5.22 ± 4.63	4.10 ± 2.88	0.388	13.41 ± 7.48	4.86 ± 3.85	< 0.001*

Values are mean ± SD or *P* values

*P < 0.05, significant difference; UT: upper trapezius; MD: medial deltoid; BB: biceps brachii; TB: triceps brachii. L: left upper limb; r: right upper limb

### Corticomuscular coupling

**Fig. 7:**
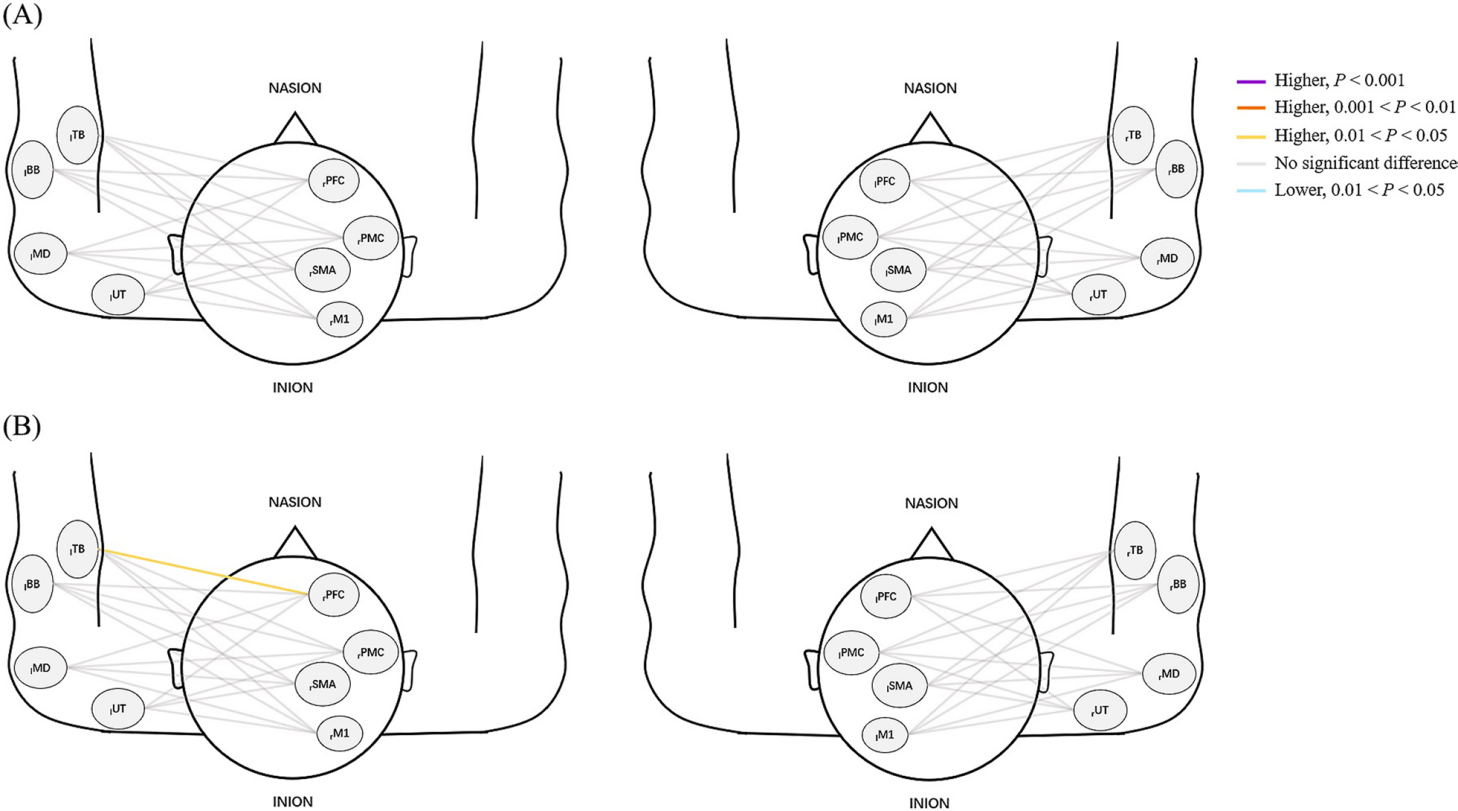
Comparison of PSI (**A**) between SCI and HC group during WCTC (**B**) between AE and WCTC among SCI group. Line color indicates the degree of significance in terms of PSI between one ROI and the contralateral muscle. Warm color represents the increased PSI value while cool color represents the decrease in PSI value. The deeper the color, the higher the significance

**Fig. 8:**
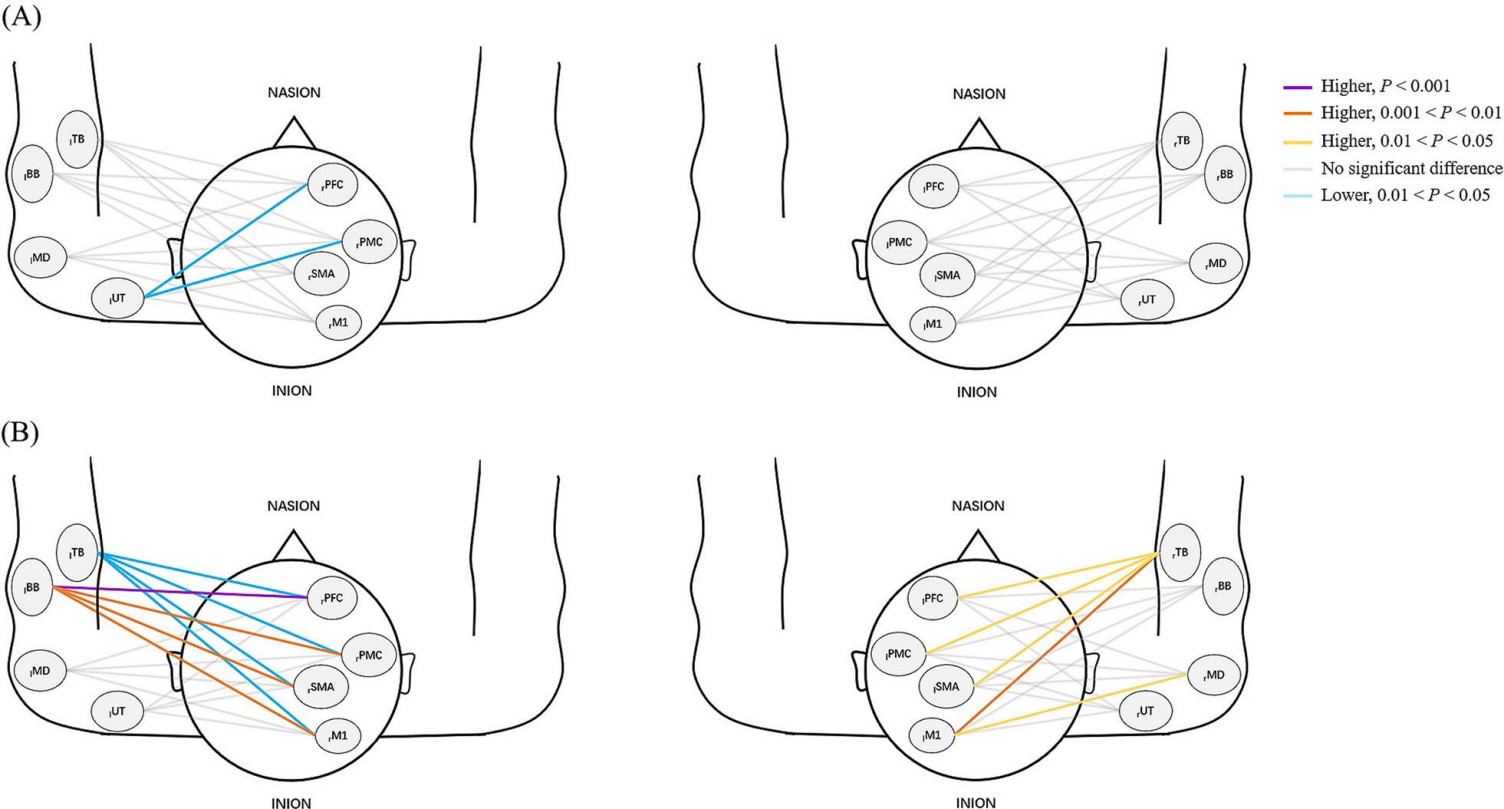
Comparison of coherence values (**A**) between SCI and HC group during WCTC (**B**) between AE and WCTC among SCI group. Line color indicates the degree of significance in terms of coherence between one ROI and the contralateral muscle. Warm color represents the increased coherence value while cool color represents the decrease in coherence value. The deeper the color, the higher the significance

The PSI and coherence values of SCI patients and HCs during WCTC are shown in Figs. 7A and 8A. Figures 7B and 8B presents the results of PSI and coherence values during WCTC and AE in patients.

#### Phase synchronization

There was no significant difference in PSI between groups. Among SCI patients, significantly higher PSI of _l_TB-_r_PFC (*P* = 0.019) was found during WCTC than AE.

#### Coherence analysis

The coherence values of _l_UT-_r_PFC (*P* = 0.019) and _l_UT-_r_PMC (*P* = 0.012) in healthy individuals were higher than that of patients. Significantly higher coherence values of _l_BB-_r_PFC (*P* < 0.001), _l_BB-_r_PMC (*P* = 0.009), _l_BB-_r_SMA (*P* = 0.008), _l_BB-_r_M1 (*P* = 0.001), _r_MD-_l_M1 (*P* = 0.025), _r_TB-_l_PFC (*P* = 0.01), _r_TB-_l_PMC (*P* = 0.04), _r_TB-_l_SMA (*P* = 0.016) and _r_TB-_l_M1 (*P* = 0.009) were found during WCTC than during AE. While performing AE, the coherence values of _l_TB-_r_PFC (*P* = 0.01), _l_TB-_r_PMC (*P* = 0.021), _l_TB-_r_SMA (*P* = 0.019) and _l_TB-_r_M1 (*P* = 0.046) were higher than WCTC.

## Discussion

This study makes a more in-depth exploration of rehabilitation for SCI, hoping that it would be beneficial to the field of neurorehabilitation. fNIRS allows us to understand the motor control information of the cortex, and sEMG primarily reflects the performance of muscles. Nevertheless, the flow of information between the brain and peripheral system is a loop, and the characteristics of corticomuscular coupling during WCTC have been poorly studied. As a supplement, coupling analysis can reflect the cooperative strength of neuromuscular motor control, and then demonstrate the connection between cerebral cortex and muscle. In this study, we observed upper limb muscle activity and cortical activity. By quantifying the phase synchronization and coherence between sEMG from upper limb muscles (upper trapezius, medial deltoid, biceps brachii, triceps brachii) and fNIRS from the contralateral ROIs (prefrontal cortex, premotor cortex, supplementary motor area, primary motor cortex), a complete understanding of WCTC in eliciting corticomuscular coupling was provided, so as to optimize rehabilitation of SCI.

We compared cortical activity, muscle activation, and corticomuscular coupling in HCs and patients during WCTC. The _l_PFC, _r_PFC, _l_PMC, _r_PMC, _l_M1, _r_M1 and _r_SMA of patients had greater activation than HCs. According to Li et al. [32], SCI patients need more mental effort than healthy individuals to carry out the same task. Sharp et al. [33] observed that the activation of multiple ROIs in patients was higher than in HC group. Changes following SCI in sensory feedback mechanisms may be one reason. Spinal cord injury leads to loss of somatosensory afferent signals, making the task more difficult for patients under the same condition. The more additional amount of effort SCI patients invest in the task, the stronger neural responses tend to take place to match the higher demands on the task, particularly in task-specific brain regions [34]. Adaptive cortical reorganization following injury may be another reason. Brain remodeling is an essential mechanism for functional recovery. Studies on electrophysiology and functional neuroimaging have shown that the human brain can be extensively reorganized [35]. Owing to the plasticity of CNS, neural circuit remodeling, and the changes of the functional map at the spinal cord and brain levels of the sensorimotor pathway may occur following SCI [36]. The altered cortical activation in patients, such as increased excitability of specific brain regions, may be related to neural remodeling. Therefore, the increased activation of the ROIs in our study is probably the phenomenon of changed sensory feedback and cerebral cortex reorganization, which promotes recovery in patients with SCI.

The FC analysis indicates coordinated activities between distinct functionally related regions. Our results showed an overall decline in FC. In the chronic phase of SCI, continuous disruption of sensorimotor pathways leads to the disintegration of functional networks, which may be manifested by changes in FC. Structural changes in the brain associated with SCI have been extensively studied and reviewed. Kaushal et al. evaluated FC in SCI using graph-based techniques [37]. The imbalanced transmission of afferent and efferent nerve impulses after injury is proposed to contribute to the decreased FC. Since function depends on structure, atrophic changes in the entire neuraxis caused by retrograde degeneration following SCI may affect functional modification. According to the hypothesis of Isa [38], large-scale network reorganization is the key to the regulation of recovery from SCI. Hence, changes in our FC results are the manifestations of the reorganization of brain involved in the recovery of autonomic function following SCI. Besides, SMA is known to select and prepare appropriate movements and is responsible for motor control [39]. An explanation for the increased FC of _l_PFC-_l_SMA, _l_PMC-_l_SMA, _l_SMA-_l_M1, and _l_SMA-_r_PFC is that after disruption of the direct pathway in the spinal cord, connection strength increases intending to provide signals to the spinal cord to control movement [40]. Our findings, together with previous results [38, 40], may suggest that patients with SCI attempt to orchestrate FC to rebuild coordination in locomotion.

In addition, our results indicated that while performing WCTC, the coherence values of _l_UT-_r_PFC and _l_UT-_r_PMC in healthy individuals were higher than that of SCI patients, and the rest showed no difference. There was no significant difference in PSI between the two groups, either. The cerebral cortex controls the movement of muscle tissue through the spinal cord and peripheral nerves, enabling the limbs to perform specific movements. Meanwhile, the movement information of the limbs affects the activities of the cerebral cortex through the afferent nerves. As a result of SCI, the properties of spinal motor neurons and the muscle fibers they innervate have been shown to change [41], resulting in an imbalance of input/output signals between the brain and the peripheral system [42]. It seems likely that the reduction of corticomuscular coupling is related to the partial interrupted corticospinal circuit [43]. This may be reflected in the differences in corticomuscular coherence values between patients and HCs. However, muscle activation was greater in the SCI group than in HCs, especially the activation of both sides of BB indicating a statistical difference. The result reflected the increased demand for the upper limb muscles among patients. Rehabilitation following SCI is primarily composed of repetitive movements over long periods to facilitate motor recovery. Such training has been proved to be related to corticospinal tract projections [44] and neural adaptations in secondary tracts [45]. Despite the cortical effort required, the increased muscle activity in SCI patients may be supported by subcortical structures including the reticulospinal and rubrospinal tracts. While given the absence of corticomuscular coupling, these auxiliary pathways may be responsible for the improvement in muscle activation, particularly for proximal upper limb muscles. Furthermore, Conway et al. [46] calculated EEG-EMG coherence, and found that corticomuscular coupling becomes more significant as muscle activity increases. Similarly, increased muscle activity results in more robust synchronization between cortex and the peripheral muscles, and more energy is required for the motor cortex to maintain steady movement [47]. Given the above, combining muscle activation, it is reasonable to speculate that SCI patients compensate for the lack of corticomuscular coupling by improving muscle activation. As a result, the phase synchronization and coherence of these two groups were similar in our study.

Physical activity is widely used for health promotion. It has a positive impact on the improvement of functional activities and the activation of motor cortex [48]. Since there are structural and functional changes in CNS and muscle tissue following injury, which exercise (WCTC or AE) is recommended as the rehabilitation method for SCI has become an urgent problem to be solved. Thus, we explored WCTC-related changes in muscle activation, cortical activity, and corticomuscular coupling versus AE. Regarding muscle activation, our results showed that both sides of BB and TB had greater activation during AE than during WCTC. WCTC is a moderate-intensity, slow-paced exercise, while AE tends to improve muscle strength with a faster rhythm than WCTC [49]. The BB and TB are antagonistic muscles. During AE, a more strenuous activity, their activation is increased by recruiting more motor units to maintain joint stability and to ensure proper movement.

From the perspective of cortical activity, the trend of the result was consistent with previous studies. There is evidence that during higher-speed motor tasks, more significant activity, and more connection strength can be observed in specific brain areas [50]. The interaction of brain regions is the key to the successful performance of complex motor tasks, and FC may change with increasing difficulty [51]. Not like WCTC, the movements of AE are more fixed and regular. Cui et al. [10] suggested that complex movements involve much broader networks than simple repetitive movements. In the process of WCTC, spatial orientations and action directions are often inconsistent, so people need to deal with the conflicting information so as to select the correct action [52]. This physiological process includes cognitive activities, and motor control, involving multiple brain regions.

Further, the effect of WCTC versus AE on the corticomuscular coupling is unclear and worth discussion. WCTC contains the motor system and CNS integration through two components: physical exercise and cognitive training. The internal and mutual effects of cerebral cortex and motor neuromuscular tissue constitute the corticomuscular coupling [53]. The increase of signal phase synchronization reflects the increased synchronization of cortical and muscle activity-related neural network oscillation [54], suggesting the increase of the coupling relationship between the systems. The results showed that PSI of _l_TB-_r_PFC during WCTC was higher than AE, indicating a closer connection between cortical and muscle activity. The coherence outcomes demonstrated that, compared with AE, coherence of _l_BB-_r_PFC, _l_BB-_r_PMC, _l_BB-_r_SMA, _l_BB-_r_M1, _r_MD-_l_M1, _r_TB-_l_PFC, _r_TB-_l_PMC, _r_TB-_l_SMA and _r_TB-_l_M1 during WCTC were higher. Considering that PSI and coherence values can reflect the relevant information of coupling between cortex and peripheral muscle in previous literature [55], the above parameters can quantify and evaluate the functional state of the neuromotor system. According to the dominance of the motor cortex over the body movement, when unilateral muscle contracts, the contralateral cortex is the central brain region controlling the movement. The fNIRS data recorded from ROI and sEMG data collected from the contralateral upper limb muscle can reflect the critical information of the brain’s driving control and the muscle’s motion response to the control intention, respectively. In this study, the greater value of PSI and coherence during WCTC may hint that the coupling of peripheral muscle and cerebral activities during WCTC in SCI patients is better than during AE.

Limitations should be acknowledged. In the present study, it remains to be determined whether the mental resources involved in WCTC (a complex, mind–body exercise) are more dispersed and are comprised of a larger scale of cortex than AE. Regarding the measuring techniques, the measurement depth of fNIRS is limited. Therefore, cortical information can only be detected, and the condition of the subcortical tissue remains unclear. Moreover, a combination of different research approaches, such as effective connectivity and Granger Causality, is recommended to better understand the underlying mechanisms of WCTC on the brain and motor neuromuscular tissue. Further longitudinal studies are needed to elaborate on the long-term effects of WCTC on rehabilitation following SCI.

## Conclusion

SCI patients require more mental resources than healthy individuals to accomplish the same task, and their FC may provide additional signals to the spinal cord for better motor control. Noteworthy is that patients compensate for the lack of corticomuscular coupling by improving muscle activation. The findings in this study can provide a complete understanding of the relationship between recovery following SCI and corticomuscular coupling. Our study also provides theoretical support for recommending WCTC as a means of rehabilitation for patients with SCI.

## Supplementary Information

**Additional file 1: Table S1.** The cortical projection coordinates of all channels in MNI space.

## Data Availability

The data that support the findings of this study are available upon reasonable request from the authors.

## Abbreviations

SCI: Spinal cord injury
HC: Healthy control
WCTC: Wheelchair Tai Chi
AE: Aerobic exercise
sEMG: Surface electromyography
fNIRS: Functional near-infrared spectroscopy
PFC: Prefrontal cortex
PMC: Premotor cortex
SMA: Supplementary motor area
M1: Primary motor cortex
HbO_2_: Oxyhemoglobin
HbR: Deoxyhemoglobin
HRF: Hemodynamic response function
FC: Functional connectivity
ROI: Region of interest
UT: Upper trapezius
MD: Medial deltoid
BB: Biceps brachii
TB: Triceps brachii
RMS: Root mean square
MVC: Maximal voluntary contraction
PSI: Phase synchronization index
